# Pentocin MQ1: A Novel, Broad-Spectrum, Pore-Forming Bacteriocin From *Lactobacillus pentosus* CS2 With Quorum Sensing Regulatory Mechanism and Biopreservative Potential

**DOI:** 10.3389/fmicb.2018.00564

**Published:** 2018-03-27

**Authors:** Samson B. Wayah, Koshy Philip

**Affiliations:** Microbiology Division, Faculty of Science, Institute of Biological Sciences, University of Malaya, Kuala Lumpur, Malaysia

**Keywords:** bacteriocin, cell-wall associated bacteriocin, quorum sensing, *Lactobacillus pentosus*, bactericidal, pore formation, broad-spectrum bacteriocin, biopreservation

## Abstract

*Micrococcus luteus, Listeria monocytogenes*, and *Bacillus cereus* are major food-borne pathogenic and spoilage bacteria. Emergence of antibiotic resistance and consumer demand for foods containing less of chemical preservatives led to a search for natural antimicrobials. A study aimed at characterizing, investigating the mechanism of action and regulation of biosynthesis and evaluating the biopreservative potential of pentocin from *Lactobacillus pentosus* CS2 was conducted. Pentocin MQ1 is a novel bacteriocin isolated from *L. pentosus* CS2 of coconut shake origin. The purification strategy involved adsorption-desorption of bacteriocin followed by RP-HPLC. It has a molecular weight of 2110.672 Da as determined by MALDI-TOF mass spectrometry and a molar extinction value of 298.82 M^−1^ cm^−1^. Pentocin MQ1 is not plasmid-borne and its biosynthesis is regulated by a quorum sensing mechanism. It has a broad spectrum of antibacterial activity, exhibited high chemical, thermal and pH stability but proved sensitive to proteolytic enzymes. It is potent against *M. luteus, B. cereus*, and *L. monocytogenes* at micromolar concentrations. It is quick-acting and exhibited a bactericidal mode of action against its targets. Target killing was mediated by pore formation. We report for the first time membrane permeabilization as a mechanism of action of the pentocin from the study against Gram-positive bacteria. Pentocin MQ1 is a cell wall-associated bacteriocin. Application of pentocin MQ1 improved the microbiological quality and extended the shelf life of fresh banana. This is the first report on the biopreservation of banana using bacteriocin. These findings place pentocin MQ1 as a potential biopreservative for further evaluation in food and medical applications.

## Introduction

Consumer's requisition for food products containing less chemical preservatives (Barbosa et al., 2017) and emergence of antibiotic resistance among pathogenic and food spoilage bacteria prompted the search for novel antimicrobials (Berendonk et al., 2015; Jia et al., 2017). Bacteriocins are an attractive class of natural antimicrobials with potential for future use as a synergistic combination with antibiotics or complete replacement of antibiotics (Behrens et al., 2017; Collins et al., 2017) and currently used chemical preservatives (Kaškoniene et al., 2017; Wiernasz et al., 2017). This is due to their ability to inhibit some drug-resistant pathogens (Mathur et al., 2017). These interesting antimicrobial peptides are of bacterial origin and are ribosomally synthesized (Langa et al., 2017). They have narrow or broad spectrum of inhibitory activity (Hanchi et al., 2017).

Bacteriocins are a highly diverse group of antimicrobial peptides. They have variations in molecular weight, inhibitory spectrum, mode of action, mechanism of biosynthesis and externalization, and self-protection mechanism (Salazar et al., 2017). They are part of the inherent defense system of bacteria and play other roles such as niche colonization, direct killing of competing strains and signaling (cross-talk and quorum sensing) within bacterial communities (Dobson et al., 2012; Inglis et al., 2013; Yang et al., 2014). Bacteriocins are commonly classified into two groups namely class I (undergo post-translational modification) and class II (unmodified). In less popular classification schemes class III (high molecular weight and heat-sensitive bacteriocins) (Alvarez-Sieiro et al., 2016) and class IV (bacteriocins with carbohydrate or lipid moieties) (Kaškoniene et al., 2017) were introduced. Bacteriocins are produced by lactic acid bacteria (LAB) and non-lactic acid bacteria (Mechoud et al., 2017). LAB bacteriocins are given more attention because they are generally recognized as safe (GRAS) facilitating their use *in situ* and *ex situ* in preservation of foods (Bali et al., 2016; Castro et al., 2017; Hu et al., 2017). Moreover, they are inactivated by gut proteases, heat-stable, active at various pH, potent even at nanomolar concentration and their biosynthetic gene cluster is often plasmid-borne, facilitating the use genetic engineering approaches in improving production (Cotter et al., 2013; Lakshminarayanan et al., 2013; Messaoudi et al., 2013; Woraprayote et al., 2016).

Biopreservation involves the use of microorganisms or their products and other natural bio-products to enhance safety and extend shelf life of food. This is achieved either by killing or reduction of the load of food spoilage microorganisms (Johnson et al., 2017; Saraoui et al., 2017). The concept of biopreservation of food has recently intensified due to growing consumer inclination toward foods containing biopreservatives or less synthetic chemical preservatives, fear of side effects of currently used chemical preservatives, demand for fresh-tasting and less processed food (Kashani et al., 2012; Woraprayote et al., 2016; Barbosa et al., 2017). Fruits and vegetables are one the major reservoirs of minerals, vitamins, and fiber and are consumed worldwide. Fresh fruits such as banana have short shelf life due to their high moisture content (Joardder et al., 2014). Preservation of banana is a huge task especially if required fresh. Moreover, eating them fresh exposes consumers to food-borne pathogens (Berger et al., 2010; Tian et al., 2012).

It has been suggested that live cultures of LAB can be used to prevent the growth of food spoilage and pathogenic bacteria on the surfaces of fruits and vegetables (Trias et al., 2008). *In situ* biopreservation of foods has not been quite successful due to two main reasons. Firstly, difficulty in getting the LAB strain adapted and established to the new environment. Secondly, difficulty in the production of an effective concentration of bacteriocin required to control spoilage and food-borne pathogens. However, a few breakthroughs have been reported. *Leuconostoc mesenteroides* was effective at inhibiting the growth of *Listeria monocytogenes* on fresh apples and lettuce (Trias et al., 2008). The Safety and shelf life of minimally processed lettuce and apples were improved by the inoculation of two strains of *Lactobacillus plantarum* (Siroli et al., 2015). A bacteriocinogenic strain of *Lactococcus lactis* inhibited the growth of yeasts and *L. monocytogenes* in minimally processed apples. Furthermore, extension of shelf life was observed (Siroli et al., 2016). Growth of *L. monocytogenes* and Salmonella on minimally processed pear was controlled by the inoculation of *Lactobacillus rhamnosus* GG (Iglesias et al., 2017). Addition of *L. rhamnosus* GG to fresh pear was an effective strategy in controlling the growth of *L. monocytogenes* (Iglesias et al., 2018).

Bacteriocin was first discovered in early 1925 when antagonistic activity was observed among strains of *Escherichia coli* (Ghazaryan et al., 2014). The discovery of colicin as the first bacteriocin was closely followed by that of nisin (1928), the first LAB bacteriocin (Shin et al., 2016). Despite the long history of LAB bacteriocins only nisin and pediocin PA-1/AcH have gained approval for preservation of selected foods (Saraniya and Jeevaratnam, 2014; Barbosa et al., 2017). The potential of bacteriocins in the biopreservation of fresh fruits or minimally processed fruits has been highly underexploited. Combined application of nisin-EDTA and chlorine was effective at reducing the surface microbial load of whole melon (Ukuku and Fett, 2002). Application of nisin, hydrogen peroxide, citric acid and sodium lactate effectively reduced the transfer of pathogens from the surface of melons to freshly cut pieces (Ukuku et al., 2005). Load of pathogens on the surfaces of minimally processed mangoes was controlled by packaging in nisin films (Barbosa et al., 2013). Enterocin AS-48 was effective at controlling contamination of raw fruits by *L. monocytogenes* (Molinos et al., 2008). Enterocin 416K1 inhibited the growth of *L. monocytogenes* on apples and grapes (Anacarso et al., 2011). The potential of preserving minimally processed papaya by applying alginate coatings containing pediocin has been demonstrated (Narsaiah et al., 2015). Biopreservation of fresh banana using bacteriocin has not been investigated.

Although *Lactobacillus pentosus* has been isolated from various sources, bacteriocinogenic strains are rare (Liu et al., 2008). A bacteriocin-producing strain of *L. pentosus* with probiotic potential has been reported (Aarti et al., 2016). Bacteriocins of *L. pentosus* origin have not been adequately studied. Pentocins have been poorly characterized and their regulatory mechanisms have not been sufficiently investigated. Their modes of action are unknown. Moreover biopreservation of fresh banana using *L. pentosus*-derived bacteriocins (commonly called pentocins) has not been studied. In this study a novel bacteriocin (pentocin MQ1) from *L. pentosus* CS2 of coconut shake origin was purified to homogeneity and characterized. Its regulatory mechanism and mode of action was investigated. Finally, its ability to preserve fresh banana was studied *in-vivo*.

## Materials and methods

### Bacterial strains and culture media

*Streptococcus pyogenes, Enterococci, Bacillus cereus, Micrococcus luteus*, and *L. lactis* were obtained from American Type Culture Collection (ATCC). *L. monocytogenes* NCTC 10890 was obtained from National Collection of Type Culture (NCTC). *Staphylococcus aureus* RF122, *Streptococcus mutans* GEJ11, *Pseudomonas aeruginosa* PA7, *Corynebacterium* spp. GH17, *E. coli* UT181, *L. plantarum* K25, and *L. pentosus* CS2 were taken from the culture collection of Microbial Biotechnology Laboratory, Division of Microbiology, Institute of Biological Science, Faculty of Science, University of Malaya, Kuala Lumpur, Malaysia. *L. plantarum* K25 and *L. pentosus* CS2 were maintained on MRS agar (Merck, Darmstadt, Germany). *S. pyogenes* ATCC 12344 and *S. mutans* GEJ11 were maintained on Todd-Hewitt agar (Difco, Le Pont de Claix, France). *M. luteus* ATCC 10240, *B. cereus* ATCC 14579, *S. aureus* RF122, *P. aeruginosa* PA7, *Corynebacterium* spp. GH17 and *E. coli* UT181 were maintained on Mueller-Hinton agar (Merck, Darmstadt, Germany). *Enterococcus faecium* ATCC BAA-2127 and *E. faecium* ATCC 349 were maintained on Tryptic soy agar (Merck, Darmstadt, Germany) while other enterococcal strains and *L. monocytogenes* NCTC 10890 were maintained on Brain-heart infusion agar (Merck, Darmstadt, Germany). *L. lactis* ATCC 11454 was maintained on M17 agar (Merck, Darmstadt, Germany) supplemented with 5% glucose (Merck, Darmstadt, Germany).

### Isolation and screening of LAB for bacteriocin production

Indigenously sourced coconut shake was inoculated into freshly prepared De man Rogose and Sharpe (MRS) broth and incubated at 37°C for 24 h. The culture was serially diluted in peptone water and LAB was isolated by growing on MRS agar plate (Merck Germany) at 37°C. MRS broth was inoculated with single colonies from a 24 h old MRS agar LAB culture and incubated aerobically at 37°C for 24 h. Screening of LAB for bacteriocin production was carried out using well diffusion assay in which cell-free supernatant (CFS) was tested for inhibitory activity against *M. luteus* ATCC 10240, *L. monocytogenes* NCTC 10890, *B. cereus* ATCC 14579, and *S. aureus* RF122. MRS agar used for well diffusion assay was supplemented with 0.1% CaCO_3_ (Friedemann Schmidt Chemical, Germany) to neutralize acidity due to organic acids.

Molecular identification of LAB was conducted by amplifying 16S rRNA gene via PCR using the universal primers 27F [5′-AGAGTTTGATC(A/C)TGGCTCAG-3′] and 1492R [5′-ACGG(C/T)TACCTTGTTACGACTT-3′]. The 16S rRNA gene was sequenced and similarity search was performed using NCBI BLAST (https://blast.ncbi.nlm.nih.gov/Blast.cgi).

### Purification, determination of molecular weight, and molar extinction coefficient

Bacteriocin was purified using adsorption-desorption approach followed by reversed-phase high performance liquid chromatography (RP-HPLC). A 24-h old culture of *L. pentosus* CS2 was subcultured in freshly prepared MRS broth in a bioreactor (Sartorius Stedim, Germany). The Bioreactor was set up (agitation at 150 rpm, temperature at 37°C) and run for 20 h after which the culture was collected and the pH was adjusted to 5.8 and allowed for 1 h. The culture was centrifuged (9,000 × g, for 20 min at 4°C) and the cell pellet re-suspended in 95% methanol (Merck, Darmstadt, Germany) with pH-value adjusted to 2. The cell suspension was stirred overnight at 4°C and subsequently centrifuged (9,000 × g for 30 min at 4°C) to obtain the supernatant which was filtered using 0.22 μm sterilized cellulose membrane (Millipore). The clear supernatant was evaporated to dryness at 40°C using a water bath and the crude bacteriocin was reconstituted in ultrapure water. Inhibitory activity was tested using well diffusion assay and crude bacteriocin was subjected to RP-HPLC containing SemiPrep RP-18e 100-10 mm column. The mobile phase consisted of two solvents: A (95% Mili-Q water Millipore, USA) and 5% acetonitrile (Merck, Germany) and B (100% acetonitrile). Elution was done using a biphasic gradient of 20–80% acetonitrile at a flow rate of 1 ml/min over 65 min. Fractions were collected and evaporated using a vacuum evaporator. Antibacterial activity of HPLC fractions were was tested. Molecular weight of the bacteriocin was determined by subjecting the active HPLC fraction to MALDI-TOF mass spectrometry. To ascertain the molar extinction coefficient, 2-fold dilutions of the bacteriocin were prepared and bacteriocin concentration was expressed in molar units. Absorbance at 280 nm was measured and a standard curve was generated from which the molar extinction coefficient was determined.

### Antibacterial spectrum

This experiment was done to ascertain the inhibitory spectrum of pentocin MQ1. Bacteriocin producer was grown in MRS broth for 20 h and CFS (400 AU/ml) was used in well diffusion assay to test antibacterial activity against selected targets. All agar plates were supplemented with 0.1% CaCO_3_ (Friedemann Schmidt Chemical, Germany) to neutralize acidity.

### Bacteriocin-cell wall association assay

An investigation was done to assess the association of pentocin MQ1 with the cell wall of its producer. Overnight broth culture of bacteriocin producer was centrifuged (9,000 × g for 20 min) at 4°C. Antibacterial activity of CFS was tested using well diffusion assay. The cell pellet was re-suspended in 95% methanol (Merck, Darmstadt, Germany) adjusted to pH = 2 and stirred overnight at 4°C on a magnetic stirrer. The cell suspension was centrifuged (9,000 × g for 30 min at 4°C) and the supernatant was filtered using a Millipore filter (0.22 μm). This was followed by evaporation of methanol on a water bath at 40°C. The cell extract was reconstituted in ultrapure water and antibacterial activity was tested.

### Bacteriocin stability test

In order to ascertain the stability of pentocin MQ1, bacteriocin preparation was exposed to different temperatures: 40, 60, and 80°C for 40 min; 100 and 121°C for 15 min. Samples were cooled to room temperature before testing antibacterial activity. Stability of bacteriocin to different enzymes (Sigma-Aldrich, St. Louis, USA) namely: proteinase K, lysozyme, pepsin, lyticase, catalase, trypsin, α-chymotrypsin, protease, proteinase, and hyaluronidase was tested. This was achieved by adding different enzyme preparations to a final enzyme concentration of 1 mg/ml and incubating for 1 h at 37°C. Inhibitory activity was tested afterwards. Bacteriocin was adjusted to various pH (2, 3, 5, 8, and 10) and incubated for 2 h at room temperature. Antibacterial activity was tested. Stability of bacteriocin upon exposure to different chemicals viz: 1% (v/v) Tween 80, 1% (v/v) Tween 20, 1% (w/v) sodium dodecyl sulfate (SDS) (Fisher scientific, New Jersey, USA) and 1% (v/v) triton X-100 was investigated. These chemicals were added to the bacteriocin and incubated for 2 h at room temperature after which antibacterial activity was tested.

### Plasmid isolation

To investigate if plasmids harbor the bacteriocin structural gene or not, plasmid isolation was carried out. This was done using easy pure® plasmid miniprep kit (TransGen Biotech, Beijing) according to manufacturer's instruction.

### Regulatory mechanism

This experiment was done to understand the regulatory system of pentocin MQ1 production. The bacteriocin was semi-purified using ammonium sulfate precipitation. This was achieved by the following procedure. An overnight MRS broth culture of the bacteriocin-producing phenotype of *L. pentosus* CS2 was centrifuged (9,000 × g, for 20 min at 4°C). The supernatant was collected and filtered using 0.22 μm sterilized cellulose membrane (Millipore) to obtain CFS. The CFS was subjected to 80% ammonium sulfate precipitation after which it was centrifuged at the same condition to obtain the precipitate. The resulting active ammonium sulfate precipitate (semi-purified bacteriocin) was dissolved in minimum ultrapure water. Another semi-purified form of the bacteriocin was obtained through hydrophobic interaction chromatography (HIC) by the following method. Active CFS from an overnight culture of the bacteriocin-producing phenotype of *L. pentosus* CS2 was subjected to HIC. Acetonitrile (Merck, Darmstadt, Germany) gradient (20, 40, 60, and 80% v/v) was used for elution of the bacteriocin adsorbed onto the surfaces of amberlite XAD-16 particles (Sigma-Aldrich, St. Louis, USA) packed in a glass column. Fractions were evaporated and antibacterial activity was determined using well diffusion assay. The active fraction from HIC was used in the next experiment. In order to investigate the regulatory mechanism of pentocin MQ1 production a bacteriocin-negative (bac^−^) phenotype was generated using the following procedure. Ten milliliters (10 ml) of fresh MRS broth was inoculated with colonies from an overnight culture of the bacteriocin-producing phenotype of *L. pentosus* CS2 and incubated at 37°C for 20 h. Cell pellet was collected by centrifugation at 2,000 rpm for 5 min. It was re-suspended in saline solution (0.85%) and washed three times at the same condition to produce a bac^−^ phenotype of *L. pentosus* CS2. Thereafter, 100 μl of this bacterial suspension was added to 900 μl of fresh MRS broth in 2 ml Eppendorf tube. This was followed by the addition of 50 μl of 0.21 μM pentocin MQ1, active ammonium sulfate precipitate (8 AU/ml), and the active fraction from HIC (8 AU/ml). These tubes were marked as “induced” while tubes that do not contain the bacteriocin were marked as “control.” All tubes were incubated at 37°C for 20 h after which 50 μl of 0.21 μM pentocin MQ1was added to the control tube. CFS from all tubes was tested for antibacterial activity. Induction of pentocin MQ1 production was said to occur if CFS from an induced tube produced inhibition zone while the control tube did not.

### Minimum inhibitory concentration

Minimum inhibitory concentration (MIC) was determined by employing the broth microdilution assay as described by Mota-Meira et al. (2000) with little modifications. Two-fold dilutions of bacteriocin were prepared in adequate media and 10 μl for pentocin MQ1 was added to 96-well microtiter plate. Overnight culture of indicator bacteria was diluted (1 × 10^8^ CFU/ml) and added to 150 μl of each bacteriocin preparation. Wells containing indicator without pentocin MQ1 were used as positive control while wells containing only the media were used as blank. Incubation was done at 37°C and optical density at 600 nm was monitored with a multiskan GO microplate reader (Multiskan GO, Thermo Scientific) over a period of 24 h. MIC was defined as the bacteriocin preparation which caused growth reduction by more than 90% compared with the positive control.

### Mode of action

#### Time-killing

This assay was done to investigate the mode and speed of action of pentocin MQ1. Indicators were grown for 10 h and centrifuged (2,000 rpm for 5 min) to collect cell pellet. Each cell pellet was re-suspended in ice-cold 5 mM sodium phosphate buffer (pH 7.2) and washed twice. The cell suspension was mixed at a ratio of 1:1 with the bacteriocin preparation (5 X MIC) and incubated at 37°C. Control consisted of bacterial suspension without the addition of bacteriocin. Experiments were done in triplicates. Growth was monitored over a period of 120 min.

#### Membrane permeabilization

Pore formation assay was done to understand the mechanism of action of pentocinMQ1. *M. luteus* was grown in Mueller-Hinton broth until OD_600nm_ = 0.45 after which 5 μM SYTOX green dye (Invitrogen, USA) was added. Ninety microliters of stained bacteria was added to MicroAmp Fast Optical 96-well reaction plate (Applied Biosystems, Life Technologies, USA). After a stable line base was attained, 10 μl of pentocin MQ1 (5 X MIC) was added to the stained bacteria. Sodium phosphate buffer (5 mM) and nisin (Sigma-Aldrich, USA) were added to stained bacteria in different wells to serve as negative and positive controls, respectively. Experiments were done in triplicates. Fluorescence as a result of binding of SYTOX green to leaked intracellular DNA was monitored using Real-Time PCR (Applied Biosystems, USA).

### Biopreservation of banana

To investigate the biopreservative potential of pentocin MQ1, a bacteriocin preparation (66.4 μM) was topically applied to mature, fresh banana samples. This was achieved by soaking sterile cotton swab in the bacteriocin preparation and gently rubbing the surfaces of banana samples with it. Some pentocin MQ1-treated banana samples were kept at ambient condition while others were refrigerated. Control samples consisted of non-pentocin MQ1-treated banana samples kept at ambient condition and others refrigerated. Samples were monitored for morphological changes. At the onset of deterioration of control samples, sterile cotton swabs were used to collect surface microflora of both control and pentocin MQ1-treated banana samples and bacterial count (CFU/ml) was measured. The experiment was allowed to proceed until the onset of deterioration of bacteriocin-treated banana samples. Shelf-life was measured. Experiments were done in triplicates.

## Results

### Isolation and screening of LAB for bacteriocin production

Sixteen different strains of LAB were isolated and identified based on 99% sequence homology. The 16S rRNA gene sequences of all isolated LAB strains were deposited in the NCBI database. *L. pentosus* CS2 with accession number MG976651 exhibited the strongest antibacterial activity and broadest antibacterial spectrum. The remaining 15 LAB strains and their accession numbers are *Lactobacillus fermentum* HFCS1(MG966463), *Weissella cibaria* BavoCS3 (MG976765), *Weissella confusa* PBCS4 (MG976757), *W. confusa* CS5 (MG976685), *W. cibaria* CS6 (MG976766), *W. confusa* CS7 (MG976686), *Lactobacillus nagelii* CS8SB (MG976652), *W. confusa* CS9 (MG980308), *L. fermentum* CS10 (MH032758), *L. fermentum* CS11 (MG966465), *L. fermentum* CS12 (MG966459), *L. fermentum* CS13 (MG966468), *L. fermentum* CS14 (MG980307), *W. cibaria* CS15 (MG982483), and *L. fermentum* CS16 (MG980073).

### Purification, determination of molecular weight, and molar extinction coefficient

**Figure 1:**
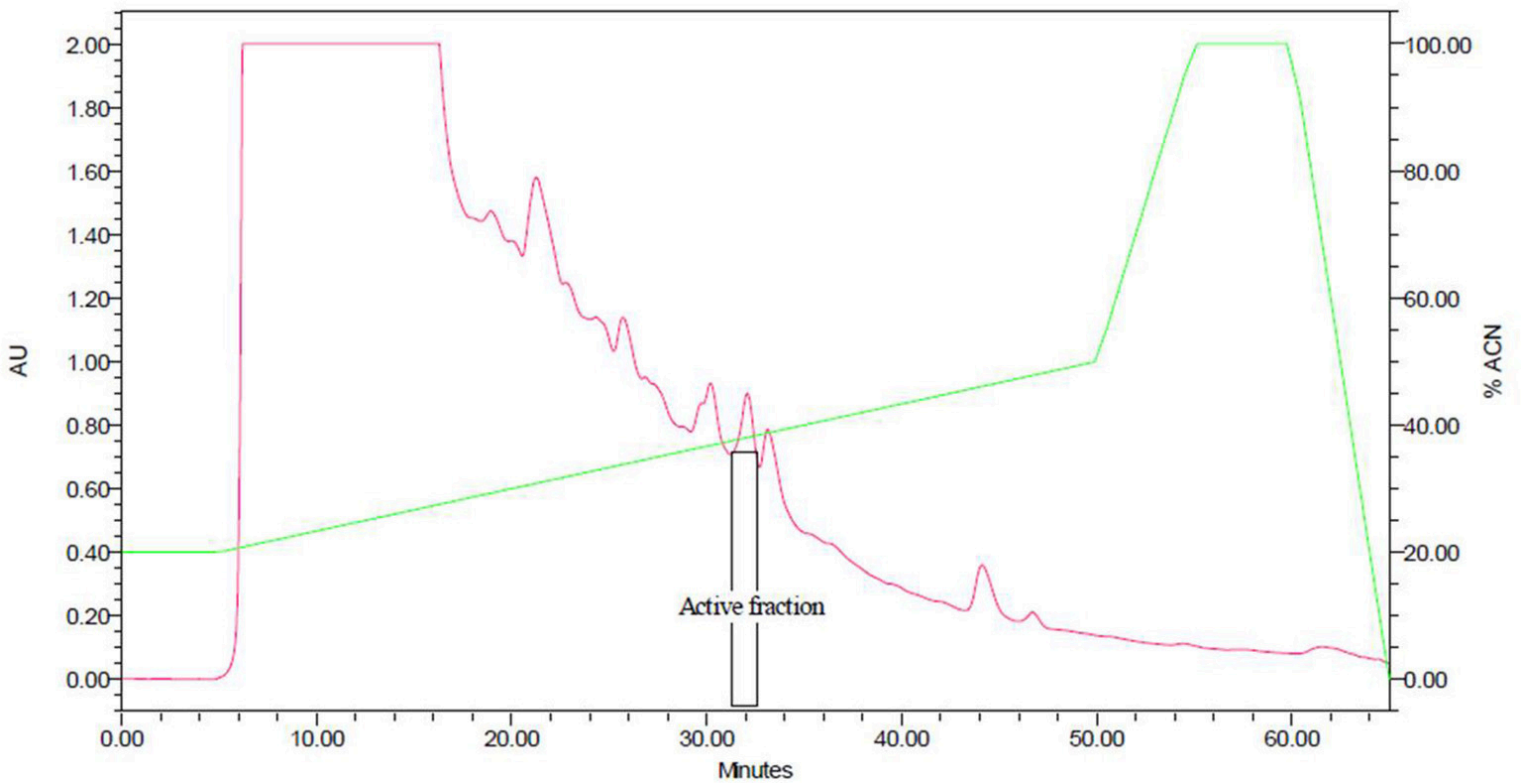
Reversed-phase HPLC chromatogram of crude cell extract from *Lactobacillus pentosus* CS2. Bacteriocin released into MRS broth was adsorbed onto the cell wall of the producer by increasing the pH of the medium to 5.8 followed by desorption from the cell wall by lowering the pH to 2. Total crude bacteriocin obtained was subjected to RP-HPLC. The vertical lines indicate the retention time.

**Figure 2:**
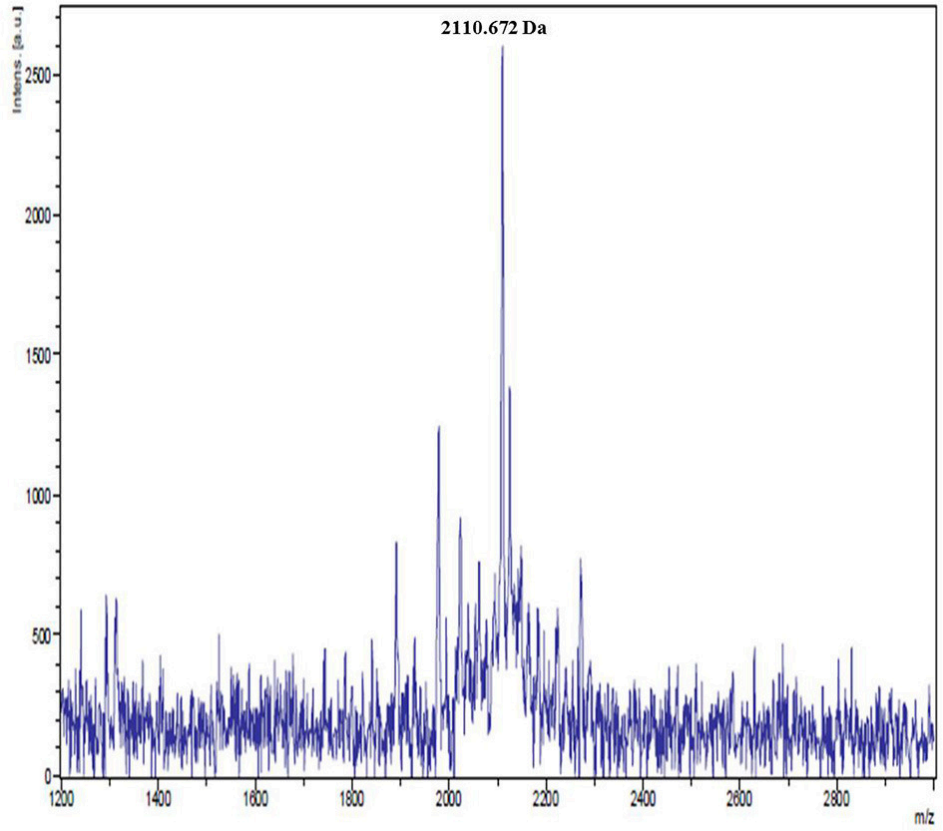
MALDI-TOF mass spectrum of purified pentocin MQ1. Purified pentocin MQ1 was subjected to MALDI-TOF mass spectrometry.

Purification of bacteriocin by a combination adsorption-desorption method and RP-HPLC proved successful. Bacteriocin was obtained at a retention time of 31–33 min (Figure 1). The purified bacteriocin resulting from RP-HPLC had a purification yield of 1.6% (Table 1). MALDI-TOF mass spectrometry revealed that the molecular weight is 2110.672 Da (Figure 2). A molar extinction coefficient of 298.82 M^−1^ cm^−1^ was obtained.

**Table 1:** Purification of bacteriocin using adsorption-desorption followed by RP-HPLC.

**Bacteriocin preparation**	**Volume (ml)**	**Activity (AU/ml)**	**Total activity (AU)**	**Yield (%)**
CFS	1,000	800	800,000	100
Cell extract	10	3,200	32,000	4.0
Purified bacteriocin	2	6,400	12,800	1.6

### Antibacterial spectrum

Pentocin MQ1 displayed strong inhibitory activity toward *L. monocytogenes* NCTC 10890, *M. luteus* ATCC 10240 and *B. cereus* ATCC 14579. It was also inhibitory albeit to a less extent toward *S. pyogenes* ATCC 12344, *S. aureus* RF122, *P. aeruginosa* PA7, *E. faecium* ATCC 19434, *E. faecium* ATCC 27270, *E. faecium* ATCC 27273, *E. faecium* ATCC BAA-2318, *E. faecium* ATCC BAA-2127, *E. faecium* ATCC 6569, *E. faecium* ATCC 25307, and *E. faecium* ATCC 349 but was not active against *S. mutans* GEJ11, *L. lactis* ATCC 11454 and *Corynebacterium* spp. GH17 (Table 2).

**Table 2:** Antibacterial Spectrum of pentocin MQ1.

**Indicator strain**	**Zone of inhibition**
*Streptococcus pyogenes* ATCC 12344	+++
*Streptococcus mutans* GEJ11	–
*Lactococcus lactis* ATCC 11454	–
*Staphylococcus aureus* RF122	+++
MRSA	+
*Listeria monocytogenes* NCTC 10890	++++
*Bacillus cereus* ATCC 14579	+++
*Pseudomonas aeruginosa* PA7	+++
*Corynebacterium spp*. GH17	–
*Escherichia coli* UT181	++
*Micrococcus luteus* ATCC 10240	++++
*Enterococcus faecium* ATCC 19434	++
*Enterococcus faecium* ATCC 27270	++
*Enterococcus faecium* ATCC 27273	++
*Enterococcus faecium* ATCC BAA-2318	++
*Enterococcus faecium* ATCC BAA-2127	++
*Enterococcus faecium* ATCC 6569	++
*Enterococcus faecium* ATCC 25307	+++
*Enterococcus faecium* ATCC 349	++
*Lactobacillus plantarum* K25	++

*++++ Inhibition zone >20mm, +++ Inhibition zone 15–20 mm, ++ Inhibition zone <15mm, – No inhibition*.

### Bacteriocin-cell wall association assay

This experiment was designed to know whether pentocin MQ1 abounds in the supernatant or cell wall of the producer. Of the total activity of 6.9 × 10^4^ AU, 66.67% (4.6 × 10^4^ AU) was detected in the cell extract while 33.33% (2.4 × 10^4^ AU) was found in the CFS (Table 3).This shows the cell-wall adhering characteristic of pentocin MQ1.

**Table 3:** Pentocin MQ1 recovered from the cell-free supernatant and cell extract of *Lactobacillus pentosus* CS2.

**Bacteriocin preparation**	**Activity (AU)**	**Activity (%)**
Cell-free supernatant	2.3 × 10^4^	33.33
Cell extract	4.6 × 10^4^	66.67
Total	6.9 × 10^4^	100.00

### Bacteriocin stability test

Stability of pentocin MQ1 under different conditions of heat, enzyme and pH are shown in Table 4. Its stability when exposed to different chemicals (1% Tween 80, Tween 20, SDS and triton X-100) are not shown in Table 4 because it retained 100% residual activity. Residual activities of 99.82, 97.99, 91.32, 90.78, and 83.11% were obtained after heating at 40, 60, 80, 100, and 121°C revealing its high thermal stability. Proteinase K, pepsin, and proteinase significantly reduced its activity (Table 4). There was a complete loss of activity when it was treated with trypsin, α-chymotrypsin, and protease. Pentocin MQ1 retained its activity after exposure to lyticase, catalase, and hyaluronidase (Table 4). pH variation had effect on its activity. It had higher activity in the pH range of 2–5 than at pH-value of 8. There was no activity at pH-value of 10 (Table 4).

**Table 4:** Stability tests for pentocin MQ1.

**Test**	**Zone of inhibition (mm)**	**Residual Activity (%)**
**HEAT**		
Control	15.95	100.00
40	15.93	99.82
60	15.73	97.99
80	15.00	91.32
100	14.94	90.78
121	14.17	83.11
**ENZYME**		
Control	15.18	100.00
Proteinase K	9.13	40.28
Lysozyme	15.18	100
Pepsin	7.87	28.19
Lyticase	14.95	97.74
Catalase	15.18	100.00
Trypsin	0.00	0.00
α-Chymotrypsin	0.00	0.00
Protease	0.00	0.00
Proteinase	10.23	51.38
Hyaluronidase	15.18	100.00
**pH**		
Control	15.80	100.00
2	16.60	107.40
3	16.53	106.76
5	16.00	101.85
8	9.10	37.96
10	0.00	0.00

### Plasmid isolation

This experiment was done to ascertain if genes encoding pentocin MQ1 production are plasmid-borne. After agarose gel electrophoresis, clear bands were observed for the 1 kb molecular ladder but no band was seen for *L. pentosus* CS2. This indicates the absence of plasmids in *L. pentosus* CS2 (Supplementary Figure 1).

### Regulatory mechanism

This assay was done to investigate the regulatory mechanism of pentocin MQ1 production by *L. pentosus* CS2. A bacteriocin-negative (bac^−^) phenotype of *L. pentosus* CS2 was produced by washing off pentocin MQ1 from the cell wall of the producer. Addition of inducible concentrations (quorum) of active ammonium sulfate precipitate, active HIC fraction and pure pentocin MQ1 to the bac^−^ phenotype of *L. pentosus* CS2 restored pentocin MQ1 production. This was made evident by the presence of inhibition zones for the induced tubes but the absence of zone of inhibition for the non-induced (control) tubes (Supplementary Figure 2).

### Minimum inhibitory concentration

Pentocin MQ1 exhibited strong inhibitory effect against *L. monocytogenes* NCTC 10890, *M. luteus* ATCC 10240, and *B. cereus* ATCC 14579. MIC-value for *M. luteus* and *L. monocytogenes* and *B. cereus* were 1.66, 1.66, and 3.32 μM, respectively.

### Mode of action

#### Time-killing

**Figure 3:**
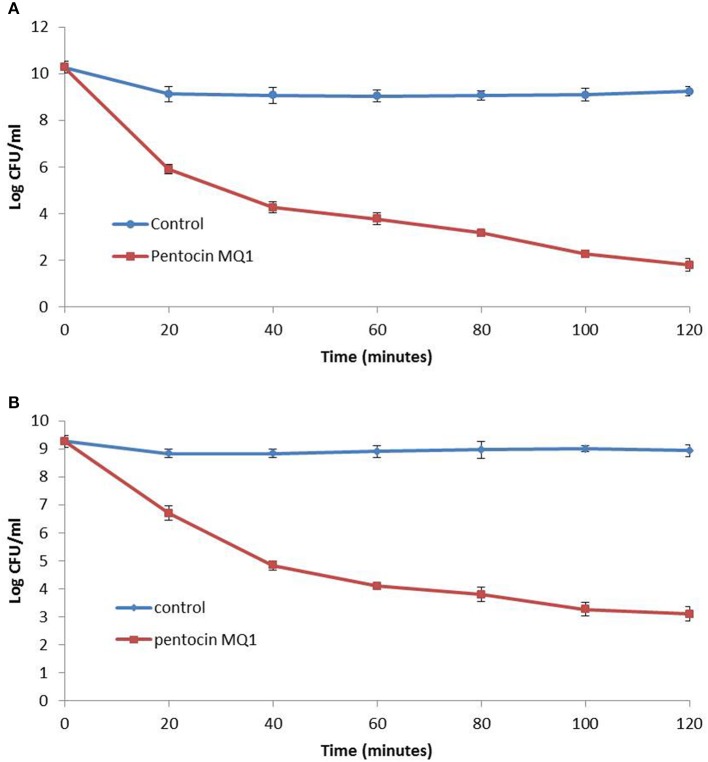
Time-killing assay for pentocin MQ1 **(A)** against *Listeria monocytogenes* NCTC 10890 **(B)** against *Bacillus cereus* ATCC 14579. Different preparations of pentocin MQ1 (5 X MIC) was added to cultures of bacterial targets. Bars indicate standard deviation.

Pentocin MQ1 caused a decline in the Log_10_ CFU/ml of *L. monocytogenes* and *B. cereus* (Figure 3). After 120 min the Log_10_ viable cell count for *L. monocytogenes* had decreased from 10.27 to 1.80 (82.47% reduction) while that of *B. cereus* had decreased from 9.27 to 3.10 (66.56% reduction).

#### Membrane permeabilization

**Figure 4:**
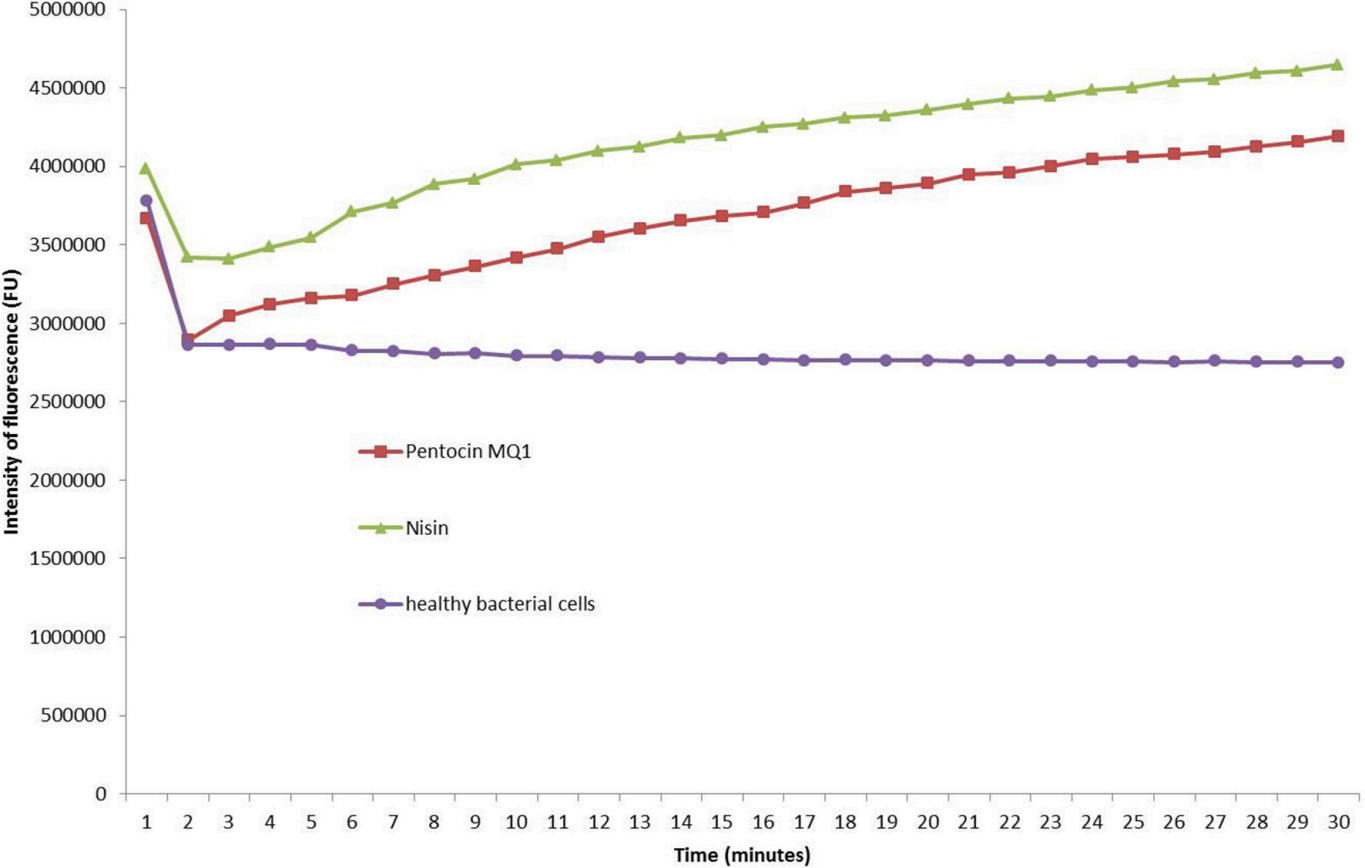
Pore-formation in the cell membrane of *Micrococcus luteus* ATCC 10240 by pentocin MQ1. Pentocin MQ1 was added to *M. luteus* stained with SYTOX green dye and increase in fluorescence as a result of leakage of intracellualr DNA was monitored using Real-Time PCR.

Treatment of *M. luteus* with pentocin MQ1 caused an increase in fluorescence intensity over the course of the study indicating pore formation. Similar observation was made for nisin although higher fluorescence intensity was observed. Fluorescence intensity of the untreated bacterial cells remained stable (Figure 4).

#### Biopreservation of banana

**Figure 5:**
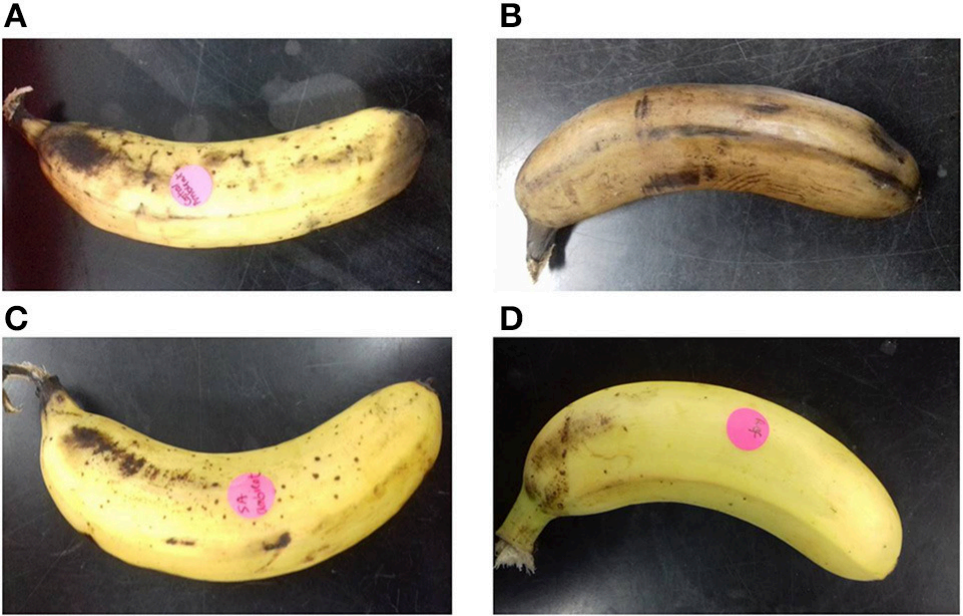
Appearance of banana after 5 days of storage at different conditions. **(A)** Nonbacteriocin-treated stored at ambient condition **(B)** nonbacteriocin-treated stored at refrigeration condition **(C)** pentocin MQ1-treated stored at ambient condition **(D)** pentocin MQ1-treated stored at refrigeration condition.

Total surface bacterial count and LAB count of 4.00 × 10^7^ CFU/ml and 2.10 × 10^3^ CFU/ml (0.005% of total bacterial count), 3.70 × 10^5^ CFU/ml and 9.40 × 10^2^ CFU/ml (0.254% of total bacterial count), 2.14 × 10^4^ CFU/ml and 1.76 × 10^3^ CFU/ml (8.22% of total bacterial count), 7.30 × 10^2^ CFU/ml and 3.20 × 10^2^ CFU/ml (43.84% of total bacterial count) were obtained for nonbacteriocin-treated sample stored at ambient condition, nonbacteriocin-treated sample stored at refrigeration condition, pentocin MQ1-treated sample stored at ambient condition and pentocin MQ1-treated sample stored at refrigeration condition, respectively (Table 5). The shelf life of nonbacteriocin-treated sample stored at ambient condition, nonbacteriocin-treated sample stored at refrigeration condition, pentocin MQ1-treated sample stored at ambient condition and pentocin MQ1-treated sample stored at refrigeration condition are 3, 5, 7, and 11 days, respectively. Total surface bacterial count and shelf had a Pearson correlation coefficient (*r*) value of −0.779 indicating a strong inverse relationship between the two parameters. An *r*-value of 0.863 was obtained for Pearson correlation analysis between percentage of LAB and shelf life of banana suggesting a strong direct relationship between the two parameters. Changes in organoleptic characteristics of nonbacteriocin-treated banana occurred much earlier than in bacteriocin-treated samples (Figure 5).These results show that treatment of banana with pentocin MQ1 extended its shelf. The microbiological quality and shelf life of pentocin MQ1-treated banana stored at refrigeration condition was better than that of pentocin MQ1-treated banana stored at ambient condition.

**Table 5:** Effect of bacteriocin application on surface bacterial count and shelf-life of banana.

**Bacteriocin**	**Bacterial count (CFU/ml) after 5 days of storage**				**Shelf-life (days)**	
	**A**		**R**		**A**	**R**
	**Total**	**LAB**	**Total**	**LAB**		
P	2.14 × 10^4^	1.76 × 10^3^	7.30 × 10^2^	3.20 × 10^2^	7	11
C	4.00 × 10^7^	2.10 × 10^3^	3.70 × 10^5^	9.40 × 10^2^	3	5

*P, pentocin MQ1; C, negative control; A, ambient condition; R, refrigerated*.

## Discussion

Bacteriocin-producing LAB confer various beneficial effects (such as improvement of quality and shelf life extension) on dairy products (Sultan et al., 2017). As such, the presence of *L. pentosus* CS2 in coconut shake suggests it has bioprotective role. Although *L. pentosus* CS2 has been isolated from vagina (Okkers et al., 1999), fermented Xuan-Wei ham (Zhang et al., 2009) and fermented shrimp (Watthanasakphuban et al., 2016), this is the first report of its isolation from coconut shake considered as a dairy product. Purification of pentocin MQ1 by sequential use of adsorption-desorption method and RP-HPLC proved successful. At low pH bacteriocins are released into the culture medium but when pH is increased to around 5.8–6.0 they become adsorbed onto the producer cells. This phenomenon was observed in this study due to the fact that no activity was detected in the CFS after the adsorption process. Adsorption-desorption approach has been used previously in the purification of some bacteriocins (Siying et al., 2000). Adsorption-desorption method has some advantages over traditional approaches such ammonium sulfate precipitation. These include reduced time of processing, purer crude bacteriocin and cheap running cost (Mu-xu and Zhi-jiang, 2009; Jia-qi et al., 2011). Based on the retention time of pentocin MQ1 which corresponds to high concentration of acetonitrile (high hydrophobicity), it can be deduced that it contains slightly more hydrophobic amino acid residues than polar or hydrophilic ones.

MALDI-TOF mass spectrometry revealed that the molecular weight of pentocin MQ1 is 2110.672 Da. There are only a few reports on purification of bacteriocin from *L. pentosus*. To date pentocins that have been successfully purified to homogeneity and molecular weight accurately determined are pentocin TV35b (3929.63 Da) (Okkers et al., 1999), pentocin 31-1 (5592.225 Da) (Zhang et al., 2009), and bacteriocin K2N7 (2.017 kDa) (Watthanasakphuban et al., 2016). The molecular weight of pentocin MQ1 does not match with any of the reported pentocins. Hence, it is a novel pentocin. Molar extinction coefficient is an important biophysical parameter that can facilitate the quantitation and future industrial application of pentocin MQ1.

Pentocin MQ1 displayed a broad spectrum of antibacterial activity. This attribute has been observed in cerein 7 (Oscáriz et al., 1999), enterocin P (Cintas et al., 1997), and enterocin LR/6 (Kumar and Srivastava, 2010). It was reported that pentocin TV35b is not inhibitory toward *B. cereus* (Okkers et al., 1999). Liu et al. (2008) also reported that pentocin 31-1 is a broad spectrum bacteriocin with inhibitory activity against *L. monocytogenes, B. cereus, S. aureus* and *E. coli*. Watthanasakphuban et al. (2016) reported that bacteriocin K2N7 has a narrow spectrum of antibacterial activity and was not inhibitory against *L. monocytogenes, B. cereus, S. aureus, E. coli*, and *E. faecium*. Pentocin MQ1 is different from pentocin TV35b, pentocin 31-1 and bacteriocin K2N7 in that in addition to the aforementioned bacterial targets it is also inhibitory against *M. luteus, S. pyogenes, P. aeruginosa*, and *E. faecium*. It also showed inhibitory activity against closely related species *L. plantarum* K25. Bacteriocins from many LAB strains have been found to inhibit the growth of both closely related and distantly-related bacterial strains (Müller et al., 2009). Broad spectrum of antibacterial activity is one of the important criteria for selection of bacteriocin for use in the biopreservation of foods (Johnson et al., 2017; Kaškoniene et al., 2017). The broad antibacterial spectrum of pentocin MQ1 well positions it as a good candidate for preservation of various types of foods.

Investigating the association of pentocin MQ1 with the cell wall of the producer is important because it can reveal whether the bacteriocin abound in the supernatant or on the cell wall. In this study more activity was detected on the cell wall than the supernatant indicating the cell wall-binding characteristic of pentocin MQ1. This finding shows that for a better recovery of pentocin MQ1 produced by *L. pentosus* CS2, an adsorption-desorption approach facilitated by pH modifications should be employed. In this study, an adsorption-desorption approach suitable for purification of pentocin MQ1 was demonstrated. Association of bacteriocin with the cell wall of the producer is thought to enhance niche competition. Cell-wall associated bacteriocins have also been described in *Lactobacillus crispatus, Streptococcus salivarius*, and *Streptococcus bovis* HC5 (Tahara and Kanatani, 1997; Mantovani et al., 2002; Barbour and Philip, 2014).

Pentocin MQ1 was highly stable to all chemical treatments investigated. This is evidenced by its retention of 100% residual antibacterial activity. It exhibited high thermal and pH stability. Higher activity was detected in the acidic pH range (2–5) while moderately alkaline pH (pH 8) caused a drastic reduction in activity. Lack of activity at pH 10 (high alkaline pH) indicates severe denaturing of pentocin MQ1. Pentocin MQ1 is a proteinaceous biomolecule due to its susceptibility to proteinases. Retention of high antibacterial activity after exposure to lyticase, catalase and hyaluronidase provides more evidence on its proteinaceous nature. Pentocin TV35b was active in the pH range of 1–10 and after heating at 60–100°C (Okkers et al., 1999). Pentocin 31-1 was active at pH 2–10 and at 60–121°C but sensitive to SDS (Liu et al., 2008). Bacteriocin K2N7 retained activity at pH 2–12 but unlike pentocin MQ1 it was inactive at 121°C (Watthanasakphuban et al., 2016). The combined attributes of chemical, pH and thermal stability of pentocin MQ1 favors its future application in food systems subjected to harsh processing conditions (Hemu et al., 2016; Yi et al., 2016). Its sensitivity to proteases is a desirable characteristic in that its chances of inhibiting beneficial components of the gut microbiota is reduced thereby, enhancing its safety (Zacharof and Lovitt, 2012; Hemu et al., 2016). Moreover, degradation of bacteriocin by proteases reduces the time of interaction between fragments of a given bacteriocin and its target thereby decreasing the possibility of resistance development (Perez et al., 2014). Its application in the treatment of gut infection would require encapsulation in nanoparticles or bioengineering to make it resistant to protease of the gut (Zhang L. et al., 2010; Arthur et al., 2014; Cavera et al., 2015).

Genetic element harboring *L. pentosus*-derived bacteriocin has not been reported. Genes encoding bacteriocin production have been detected on plasmids and chromosomes (Garcia et al., 2010). Absence of plasmids in *L. pentosus* CS2 suggests that genes encoding pentocin MQ1 production are chromosome-borne. It is thought that chromosome-encoded bacteriocin genes are more stable than plasmid-encoded bacteriocin genes because plasmids, being small and mobile genetic elements can be lost by leaking out of bacterial cells (Sengupta and Austin, 2011). Hence, bacteriocin-producing LAB strains harboring chromosome-borne bacteriocin genes have an edge over those with plasmid-borne bacteriocin genes. Thus, *L. pentosus* CS2 is genetically stable. Chromosome-borne bacteriocins include enterocin A (Aymerich et al., 1996) and ABP-118 (Flynn et al., 2002) and acidocins LF221 (Majhenič et al., 2003).

Pentocin MQ1 production was restored when active ammonium sulfate precipitate, active HIC fraction and pure pentocin MQ 1 were added separately to the bac^−^ phenotype of *L. pentosus* CS2. This shows that pentocin MQ1 production is auto-inducible suggesting its regulation by a two-component quorum sensing mechanism involving an inducing peptide (the bacteriocin), a histidine protein kinase (HPK) and a response regulator (RR). Pentocin MQ1 acts as the inducing peptide. Upon attaining an inducible concentration (quorum) the bacteriocin binds to HPK and activates it. Activated HPK phosphorylates RR which in turn binds to the promoter of bacteriocin genes and upregulates bacteriocin production (Chanos and Mygind, 2016). Nisin production is regulated by a similar mechanism (Dobson et al., 2012). Pentocin 31-1 production is also controlled by quorum sensing (Zhang et al., 2012). Regulation of bacteriocin production via quorum sensing mechanism is commonly found among class II bacteriocins. (Straume et al., 2007; Di Cagno et al., 2010, 2011).

Pentocin MQ1 was strongly inhibitory against *L. monocytogenes* NCTC 10890, *M. luteus* ATCC 10240, and *B. cereus* ATCC14579 at micromolar concentrations. High activity at low concentration is a desirable property of natural biopreservatives (Bali et al., 2016). MIC-values for nisin A against *Micrococcus* spp. and *Bacillus* spp. (Mota-Meira et al., 2000) are lower than that of pentocin MQ1. However, the broad antibacterial spectrum of pentocin MQ1 suggests wide food and medical applications. Pentocin MQ1 exhibits a bactericidal mode of action against *L. monocytogenes* and *B. cereus*. After 120 min the viable cell count for *L. monocytogenes* and *B. cereus* had been reduced significantly. This shows the quick-acting characteristic of pentocin MQ1 against these pathogens. Pentocin 31-1 was also shown to exert a bactericidal effect against *L. monocytogenes* (Liu et al., 2008). Pentocin TV35b had a bactericidal activity against *Listeria innocua* (Okkers et al., 1999).

The two main modes of action of LAB bacteriocins against Gram-positive bacteria are pore formation and inhibition of cell wall synthesis (Cotter et al., 2013). Inhibition of cell wall biosynthesis of *M. luteus* by salivaricin B did not cause an increase in fluorescence within 30 min. But within the same time frame nisin, a known pore former caused an increase in fluorescence intensity (Barbour et al., 2016). This implies that pore formation is a more rapid killing mechanism. Since increase in fluorescence intensity was observed for the pentocin MQ1-treated *M. luteus* within 30 min it can be concluded that it was due to membrane permeabilization. Pentocin MQ1 caused membrane permeabilization of *M. luteus* leading to leakage of intracellular DNA and consequently death of the bacteria. This is the first report on membrane permeabilization as a mechanism of action of *L. pentosus*-derived bacteriocins. It is thought that pore formation also led to loss of other valuable intracellular molecules such as ATP contributing to the rapid death of the bacterial target. Pentocin MQ1 was quick-acting against its target. It is thought that resistance to a quick-acting antimicrobial agent is less likely to occur compared to a slow-acting one. Pore formation has been reported for several LAB bacteriocins (Perez et al., 2014; Snyder and Worobo, 2014).

Banana is one of the most consumed fruit in the tropics and subtropics (Huang et al., 2014). It is a good source of antioxidants, carbohydrates, calcium, and potassium (Mohapatra et al., 2011). As a perishable and climacteric crop it has a short shelf life. Preserving fresh banana is quite challenging (Mohapatra et al., 2010). Various chemical and physical approaches are employed in the preservation of banana (Zaman et al., 2007; Kudachikar et al., 2011; Mohapatra et al., 2011). However, consumer inclination toward foods containing biopreservatives and less chemical preservatives triggered the search for natural products that can be used for biopreservation (Barbosa et al., 2017). In a recent study, combined application of phenylurea and gibberellins was effective at extending the shelf life of banana (Huang et al., 2014). Although the bioprotective capabilities of several bacteriocins have been reported (Galvez et al., 2008; Abriouel et al., 2010; Bhatia et al., 2016), no report has been made for banana. Only one report has been made on biopreservation of food using pentocin. In that study, the potential of pentocin 31-1 for preserving pork meat was demonstrated (Zhang J. et al., 2010). Topical application of pentocin MQ1 extended the shelf of banana in this study. Shelf life extension was due to decrease in total bacterial count and increase in the percentage of LAB compared to the other microflora (Table 5). It can be deduced that pentocin MQ1 decreased the population of pathogenic and spoilage bacteria on the surface of banana. Moreover, it had a positive effect on the population dynamics of the surface microflora such that decrease in spoilage bacteria enhanced the growth of beneficial LAB strains leading to shelf life extension. These results reveal the biopreservative potential of pentocin MQ1. Furthermore, bacteriocin treatment and refrigeration had a synergistic effect on the microbiological quality of banana resulting in extension of shelf life. These findings pave the way for future *ex situ* application of pentocin MQ1 in the biopreservation of banana.

In conclusion, this is the first report on the presence of bacteriocinogenic strain of *L. pentosus* in coconut shake. *L. pentosus* CS2 produces a novel bacteriocin (pentocin MQ1) with a broad spectrum of antibacterial activity, high chemical, thermal and pH stability but sensitive to proteolytic enzymes. It is cell-wall associated and possesses a bactericidal mode of action. Pentocin MQ1 acted against its target through pore formation. Genes encoding pentocin MQ1 production are not plasmid-borne. Its biosynthesis is regulated by a quorum sensing mechanism. Its ability to preserve fresh banana was demonstrated in this study. The characteristics of pentocin MQ1 show its potential for the preservation of food.

## Author contributions

KP designed, supervised execution of the experiments, and wrote the manuscript; KP also edited the manuscript; SW designed the experiments, performed it, and wrote the manuscript.

### Conflict of interest statement

The authors declare that the research was conducted in the absence of any commercial or financial relationships that could be construed as a potential conflict of interest.

## Abbreviations

CFS: Cell-free supernatant
FU: Fluorescence unit
HIC: Hydrophobic interaction chromatography
LAB: Lactic acid bacteria
MALDI-TOF: Matrix-assisted laser desorption ionization time-of-flight
MIC: Minimum inhibitory concentration
MRS: De Man, Rogosa and Sharpe
RP-HPLC: Reversed-phase high performance liquid chromatography
SDS: Sodium dodecyl sulfate.
