# Supplementary figures and images for: Pentocin MQ1: A Novel, Broad-Spectrum, Pore-Forming Bacteriocin From Lactobacillus pentosus CS2 With Quorum Sensing Regulatory Mechanism and Biopreservative Potential
Source: Front Microbiol. 2018 Mar 27;9:564. doi: 10.3389/fmicb.2018.00564 (PMC5880951; doi:10.3389/fmicb.2018.00564)

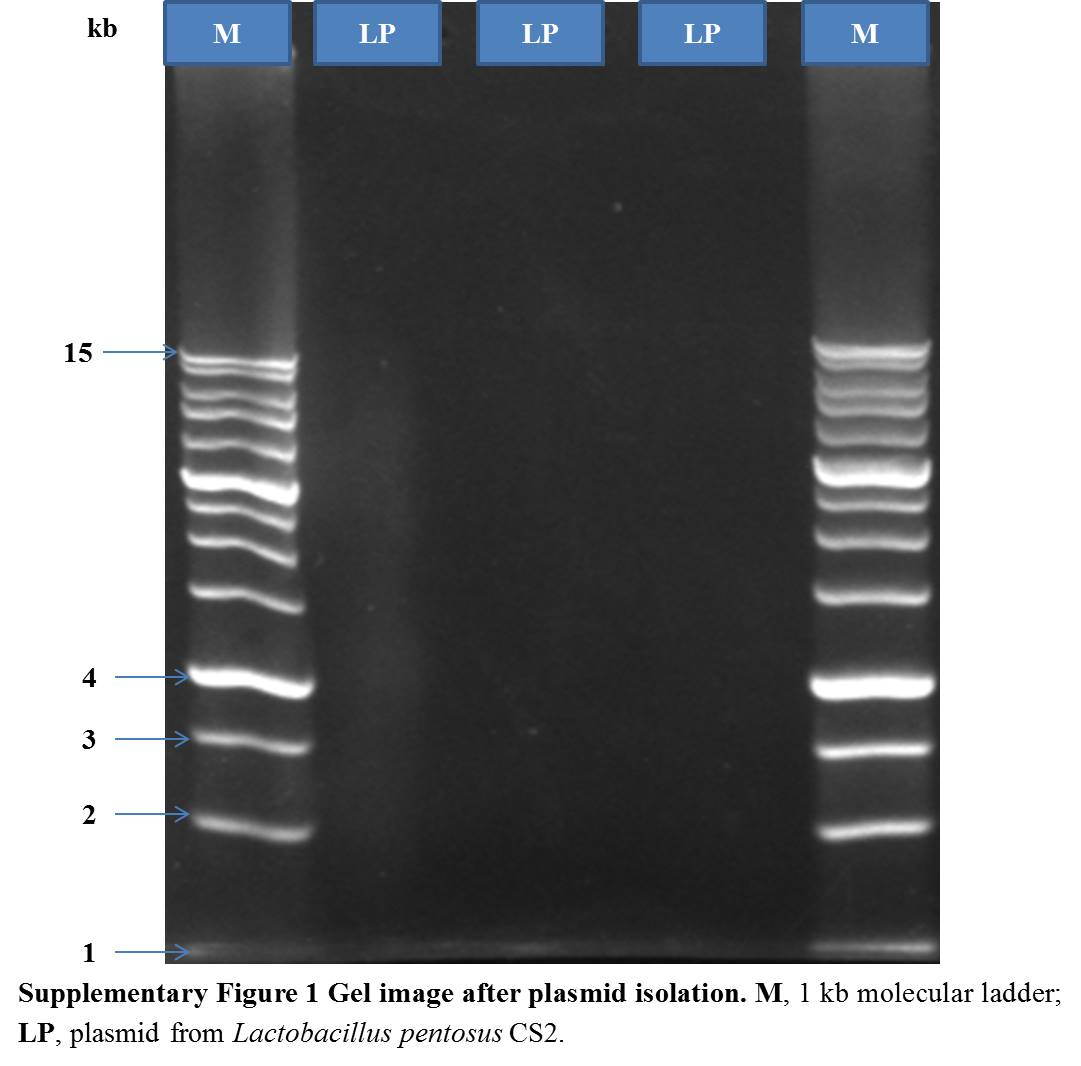

Supplement: Supplementary file 1 [file Image1.JPEG]

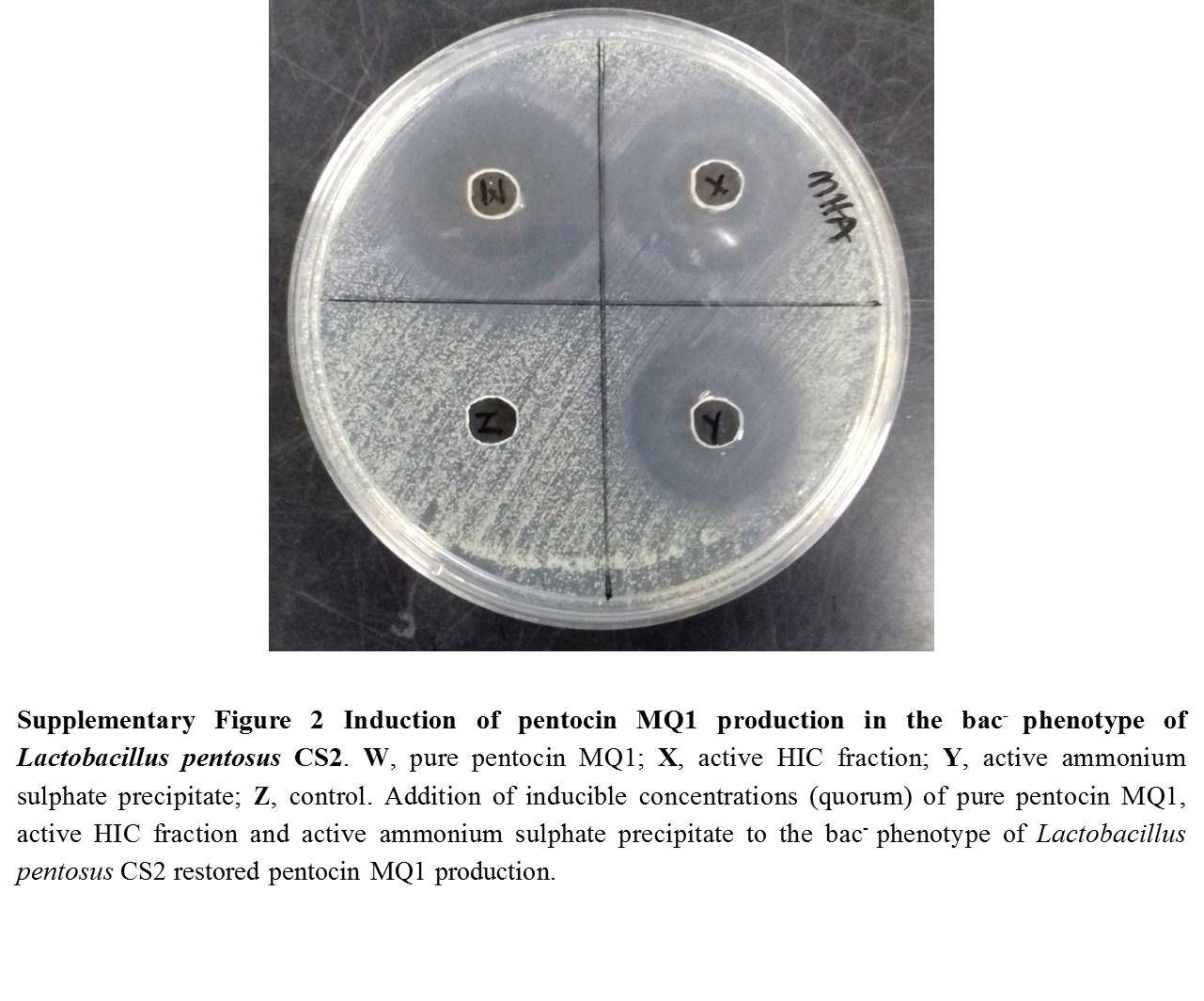

Supplement: Supplementary file 2 [file Image2.JPEG]
